# Characterization of severe *Plasmodium falciparum* malaria patients in an Angolan general ICU

**DOI:** 10.1186/1475-2875-9-S2-P52

**Published:** 2010-10-20

**Authors:** Esmael Tomás, E Filipe, E Viegas, A Sá, F Silva, L Antunes, E Lafuente

**Affiliations:** 1 Clínica Sagrada Esperança, ICU, Angola; 2 Centro Hospitalar Tamega Sousa, ICU, Portugal

## Introduction

Malaria is the most important human parasitic disease, causing an estimated 500 million cases and more than 1 million deaths annually. *Plasmodium falciparum* is responsible for the most serious form of the disease with a significant mortality rate.

## Objective

To characterize severe *Plasmodium falciparum* malaria patterns in patients admitted to an Angolan general ICU.

## Methods

A retrospective study based on medical records of adult patients with severe *Plasmodium falciparum* malaria admitted between January 2006 and December 2008 at an Angolan University-affiliated teaching hospital. We collected data on demographics, malaria-related immunity status, clinical presentation, WHO malaria severity criteria, laboratory findings and outcome. Continuous data were analyzed with Students t-test. A *P* of less than 0.05 was regarded to be significant.

## Results

Out of 114 patients admitted with diagnosis of malaria, we enrolled 56 patients. Forty-four (79%) were males. The mean age was 43.0±12.9. Twenty-eight (50%) were nonimmune and only two were adherent to chemoprophylaxis, but reported taking it incorrectly. Fifty-two percent were admitted during the second trimester. The mean APACHE II was 15.4±8.7 with a mean predicted dead rate of 25.9%. The mean SOFA on admission was 7.6±3.6. Fever (82%) followed by headache (41%) and gastrointestinal symptoms (36%) were the most common symptoms on admission and jaundice (61 %) the most common sign. The mean duration of symptoms prior to presentation at the ED was 6.1 ±4.3 days. Malaria diagnosis was confirmed within 24 hours of admission to our hospital In all the cases. Twelve patients presented 2 or more WHO severity major criteria. Forty-one patients were treated with quinine and twelve with artemether. Nineteen patients (34%) required ventilatory support, twenty (36%) intermittent hemodialysis and twelve (21%) vasopressor support. The mean ICU length of stay was 5.5±3.8 days. The 2-day mortality rate and total ICU mortality rate recorded was 10.7% and 37.5%, respectively.

**Table 1:** Characteristics of survival and nonsurvival groups

	Survival (n=35)	Non-survival (n=21)	p
Age, Mean ± SD	43.7 ± 12.5	42.0 ± 14.0	0.66
APACHE 11, Mean ± SD	12.1 ± 6.2	21.0 ± 9.4	<0.0001
SOFA on admission (total), Mean ± SD	6.5 ± 3.3	9.6 ± 3.5	0.0013
Non-Immune, n(%)	18(51.4)	10 (47.6)	0.79
Two or more WHO severity criteria, n(%)	1 (0.3)	11 (52.4)	<0.0001
Platelets (x103/mm^3^), Mean ± SD	85.8 ± 86.3	65.3 ± 57.3	0.35
Bilirubin (mg/dl), Mean ± SD	4.8 ± 4.7	6.9 ± 7.2	0.08
Creatinine (mg/dl), Mean ± SD	3.6 ± 3.1 5	3.9 ± 2.6	0.67
C-reactive protein (mg/l), Mean ± SD	118.0 ± 87.5	140.0 ± 103.0	0.45
Arterial lactates (mmol/l), Mean ± SD	3.4 ± 2.89	5.4 ± 4.6	0.019
Parasitemia (x10^3^parasites/mm^3^), Mean ± SD	30.5 ± 28.8	27.8 ± 36.7	0.73

## Conclusions

In this review, the criteria usually pointed as predictors of a poor outcome on sepsis cases were found to have statistical significance in malaria-related deaths.
